# Bmi-1 induces radioresistance in MCF-7 mammary carcinoma cells

**DOI:** 10.3892/or.2011.1615

**Published:** 2011-12-30

**Authors:** ZHI-GANG LIU, LI LIU, LI-HUA XU, WEI YI, YA-LAN TAO, ZI-WEI TU, MAN-ZHI LI, MU-SHENG ZENG, YUN-FEI XIA

**Affiliations:** 1 State Key Laboratory of Oncology in Southern China; 2 Department of Radiation Oncology, Sun Yat-sen University Cancer Center, Guangzhou 510060, P.R. China; 3 Department of Experimental Research, Sun Yat-sen University Cancer Center, Guangzhou 510060, P.R. China

**Keywords:** Bmi-1, radioresistance, mammary carcinoma cells

## Abstract

Bmi-1, a member of the polycomb family, it is involved in self renewal of stem cells and functions as an oncogene in many malignant human cancer types. Recent studies have demonstrated that Bmi-1 is a predictive factor for poor patient prognosis. However, the underlying mechanisms of radioresistance mediated by Bmi-1 are poorly understood. In this study, the dose-survival relationship was analyzed using a clonogenic survival assay and combined radiation treatment with Bmi-1 overexpression or silencing. DNA double-strand break (DSB) and repair was assessed by immunofluorescence staining of γH2AX foci. In addition, mitochondrial membrane potential was detected between Bmi-1 knockdown and control MCF-7 cells after irradiation. Apoptosis and cell cycle were evaluated by flow cytometry. We found that exposure of MCF-7 cells overexpressing Bmi-1 to ionizing radiation resulted in dramatically enhanced survival relative to control cells, whereas cells with silenced Bmi-1 showed markedly reduced survival. Bmi-1 inhibition significantly increased DSBs and decreased DSB repair. Furthermore, Bmi-1 knockdown induced loss of mitochondrial membrane potential and enhanced apoptosis by up-regulating p53, p21, Bax expression and down-regulating p-AKT and Bcl-2 expression. These results indicate that Bmi-1 may play an important role in radiosensitivity, and the suppression of its expression might be a potential therapeutic target for breast cancer.

## Introduction

Polycomb group (PcG) proteins, which are known to maintain the silenced state of homeotic genes, are important for constituting a cellular memory system responsible for maintaining the epigenetic status of target genes throughout the lifetime of the organism (1,2). PcG proteins play a crucial role in many physiological processes, such as germline development, cell differentiation, pluripotency and stem cell self-renewal (3,4). PcG proteins form transcriptional repressor modules that functionally can be divided into at least three distinct classes of complexes: polycomb repression complex 1 (PRC1), including RING1A, HPC1-3, HPH1-3, and Bmi-1, is to maintain repression; PRC2, with the core proteins EZH2, EED, and SUZ12, is to inhibit gene expression directly. Both PRC1 and PRC2 members have been found involved in malignant transformation and tumor development in various hematological and epithelial cancers (5).

B-cell-specific moloney murine leukemia virus integration site 1 (Bmi-1) is a member of PRC1 that was initially identified as an oncogene involved in the development of mouse pre B-cell lymphoma cooperating with c-Myc (6,7). Many studies have demonstrated that Bmi-1 protein regulates the INK4a/ARF locus, which encodes the two tumor suppressors, p16^INK4a^ and p14^ARF^ (p19^ARF^ in mouse), which act in pRb and p53 cell cycle control pathways, respectively (8,9). Bmi-1 promotes cellular proliferation by repression the expression of the INK4a/ARF locus (9). Moreover, overexpression of Bmi-1 in epithelial cells could induce human telomerase reverse transcriptase activity, which is associated with cell immortalization (10,11). It has also been shown that Bmi-1 overexpression together with H-Ras promotes human mammary epithelial cell (HMEC) transformation and breast oncogenesis (12). Interestingly, Bmi-1 has been recently shown to play a crucial role in self renewal of hematopoietic and neural stem cells and leukemic stem cells (13–15). Previous studies also showed that Bmi-1 plays important roles in regulating self-renewal of normal and tumorigenic human mammary stem cells (16).

In clinical study, overexpression of Bmi-1 has been correlated with cancer susceptibility and poor prognosis in several human cancers, including non-small cell lung cancer (17), gastric carcinoma (18), hepatocellular carcinoma (19), acute myeloid leukemia (20), breast cancer (21), nasopharyngeal carcinoma (22), and bladder cancer (23). Furthermore, Bmi-1-associated gene expression pathway, which is 11 gene Bmi-1 stem cell signature, is a powerful predictor of a short interval to distant metastasis, highly malignant clinical course of disease progression, and high likehood of therapy failure in multiple types of human cancer (24). Epithelial-mesenchymal transition (EMT), epithelial cells acquire mesenchymal-like properties, which increase cell motility, and EMT generates cells with properties of stem cells (25). Bmi-1 is essential in EMT process (22,26,27), and EMT also mediates radioresistance in human cancer cells (28,29). Our previous study demonstrated that Bmi-1 promotes the invasion and metastasis of human breast cancer and predicts poor survival, the inhibition of Bmi-1 reverses the expression of EMT markers and inhibits the Akt/GSK3β/Snail pathway (30). These observations led us to hypothesize that abrogation of Bmi-1 expression could be a potential therapeutic strategy against human cancers. We hypothesized that Bmi-1 inhibition combined radiotherapy could induce synergistic effect on MCF-7 tumor cells. We tested this hypothesis by evaluating the effects of Bmi-1 inhibition by shRNA on DNA damage, apoptosis, mitochondrial membrane potential (ΔΨm) and apoptosis related protein for the purpose of a more improved cancer therapy.

## Materials and methods

### Cell culture

MCF-7 (human breast cancer) cell line was obtained from the American Type Culture Collection (ATCC, Manassas, VA) and incubated in DMEM (Invitrogen, Carlsbad, CA) supplemented with 10% fetal bovine serum (FBS; Hyclone, Logan, UT), penicillin (100 units/ml), and streptomycin (100 units/ml) at 37°C in humidified 5% CO_2_ incubator.

### Generation of stable Bmi-1 overexpression and Bmi-1 knockdown (KD) cell lines

Retroviral vector pMSCV-Bmi-1 and Bmi-1 short hairpin RNA (shRNA) was previously described (22, 28). Retroviruses were generated by transients transfection as described (31). Bmi-1 gene was introduced into MCF-7 cells by infecting cells with a retroviral vector pMSCV-Bmi-1 or pMSCV-Bmi-1-shRNA (knockdown, KD). Control cells were infected with the empty retroviral vector pMSCV. Retrovirus-infected were selected and maintained in 0.5 μg/ml puromycin for 7 days and used as stable cells.

### Radiation clonogenic survival assay

Cells were seeded in triplicate into 60-mm culture dishes in a range of 100–10,000 cells per dish, depending on the radiation dose that the cells received and the test condition, so as to yield 0–100 colonies per dish. Cells were then irradiated with a single dose of X ionizing radiation (irradiation rate of 104.93 cGy/min at 210 kV and 12 mA) (32), including 0, 1, 2, 4 and 6 Gy. Immediately after irradiation, the treated cells were cultured in a 37°C, 5% CO_2_ incubator for 10–14 days. Individual colonies (>50 cells per colony) were fixed with methanol and stained with crystal violet. Plating efficiency (PE) and survival fractions (SF) were calculated. Survival curves were fitted and analyzed using linear-quadratic model [S=exp(−αD−βD^2^)] by GraphPad Prism software (version 4.0, GraphPad Prism software, San Diego, CA). The radiation sensitizing enhancement ratio (SER) by Bmi-1 inhibition was calculated using the following formula: SER=(SF_2_ of MCF-7 infected by control vector)/(SF_2_ of Bmi-1-overexpressing MCF-7 or Bmi-1-silencing MCF-7). SER=1 suggests an additive radiation effect and SER >1, a supra-additive effect as against a sub-additive effect in the case of SER <1.

### Immunofluorescence staining

Cells were seeded on coverslips in 6-well plates and allowed to grow overnight. Cells were irradiated with a single dose of 0, 1, 2, 4 and 6 Gy. After 15 min, cells was washed and fixed with 4% paraformaldehyde. Or cells were fixed at designed measuring time (0, 15, 30 min, 1, 3, 6 and 20 h) after irradiated with a single dose of 5 Gy. Then, cells were rinsed in phosphate-buffered saline (PBS). After blocking in 10% normal blocking serum at room temperature for 10 min, slides were incubated with γH2AX antibody (Cell Signaling Technology, Beverly, MA) at 4°C overnight and then incubated with goat anti-rabbit IgG conjugated with FITC (Molecular Probe, Carlsbad, CA). Slides mounted with Prolong Gold antifade reagent with DAPI (Molecular Probe) and examined by fluorescence microscopy (Carl Zeiss Axioskop 2, Thornwood, NY). Cells were judged as ‘positive’ for γH2AX foci when they displayed 10 or more discrete dots of brightness. For quantitation of foci, a minimum of 100 cells were analyzed for each time point. All data points represent mean ± SD of three experiments.

### FACS analysis

After 8 Gy irradiation, cells were harvested at designed measuring time (0, 3, 5 and 7 days) and fixed with 70% ice-cold ethanol. Cells were treated with RNase A (50 μg/ml, Sigma-Aldrich, St. Louis, MO) and stained with propidiumiodide (PI, 50 μg/ml, Sigma-Aldrich). The fluorescence of DNA-bound PI in cells was measured with FACSCalibur Flow Cytometer (Becton-Dickinson, Franklin Lakes, CA).

### Measurement of mitochondrial membrane potential (ΔΨ m

Mitochondrial membrane potential was measured using the mitochondrial-specific dual-fluorescence probe, JC-1, based on the method previously described (33). Cells were irradiated with 0 or 5 Gy. After 24 h, cells were incubated with the 10 μg/ml JC-1 for 10 min followed by two wash with PBS. A CytoFluor plate reader (excitation wavelength 485 nm, slit width 20 nm) was used to monitor the fluorescence intensities for the monomer and the aggregated JC-1 molecules (emission wavelength 520 nm, slit width of 25 nm, and 580 nm, slit width of 30 nm, respectively). Results were expressed as fluorescence ratio (580/530 nm).

### Western blot analysis

Cells were washed in cold phosphate-buffered saline (PBS) and lysed in a RIPA buffer containing 50 mM Tris-HCl pH 7.4, 150 mM NaCl, 1 mM PMSF, 1 mM EDTA, 5 μg/ml aprotinin, 5 μg/ml leupeptin, 1% Triton X-100, 1% sodium deoxycholate and 0.1% SDS. The protein concentration was determined by the Bradford dye method (Bio-Rad Laboratories, Hercules, CA). Equal amounts of cell extract were subjected to electrophoresis in 10% or 15% SDS-PAGE and transferred to polyvinylidene difluoride membranes (Amersham Pharmacia Biotech, Piscataway, NJ). The membrane was probed with primary antibody overnight, subsequently incubated with horseradish peroxidase-conjugated anti-rabbit or anti-mouse immunoglobulin G (1:2000; Santa Cruz Biotechnology, Santa Cruz, CA) and detected by enhanced chemiluminescence (Amersham Pharmacia Biotech) according to the manufacturer’s suggested protocols. Mouse anti-Bmi-1 (F6) antibody was from Upstate Biotechnical (Lake Placid, NY), rabbit anti-phosphorylated Akt (Ser473) antibody were from Cell Signaling, and mouse anti-p53 (DO-1), mouse anti-p21 (F-5), mouse anti-Bax (6A7), Bcl-2 (C-2), mouse anti-α-tubulin antibodies were from Santa Cruz Biotechnology.

### Statistical analysis

Statistical analysis was performed using Student’s t-test or ANOVA test by SPSS 12.0 software (Abbott Laboratories, North Chicago, IL). Differences were considered statistically significant at P<0.05. We performed each study at least three times under identical conditions.

## Results

### Bmi-1 expression is associated with radiation response of MCF-7 cells

**Figure 1:**
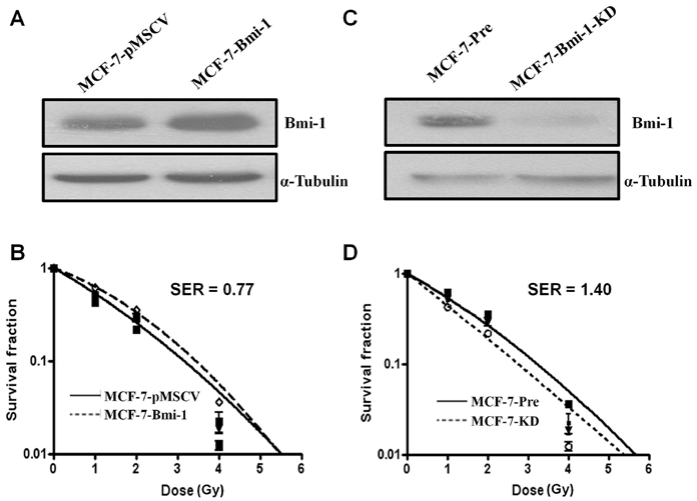
Bmi-1 regulates radiosensitivity in MCF-7 cell line. (A) Western blot analysis performed on pMSCV control vector and expressing Bmi-1 using anti-Bmi-1, and anti-α-tubulin antibodies. (B) Increased expression of Bmi-1 enhances resistance to radiotherapy. P=0.007; ANOVA test (C) Western blot analysis performed on shBmi-1 or vector cells using anti-Bmi-1, and anti-α-tubulin antibodies. (D) Down-regulation of Bmi-1 increase the sensitivity of MCF-7 cells to irradiation. P<0.001; ANOVA test.

We first studied whether Bmi-1 overexpression was correlated with radiosensitivity of MCF-7 cells. For this purpose, we infected MCF-7 cells with a retroviral vector expressing Bmi-1 or pMSCV control vector. After puromycin selection, overexpression of Bmi-1 was confirmed by Western blot analysis (Fig. 1A). By classical radiation clonogenic survival assay, we found that Bmi-1-overexpressing cells (MCF-7-Bmi-1) had more radioresistance and higher survival than pMSCV vector infected (MCF-7-pMSCV) cells (F=4.183, P=0.007; ANOVA test) (Fig. 1B). Statistical analysis of the survival parameters calculated using the linear-quadratic model revealed that there was a significant difference in the α value, characterizing the initial slope of the curving radiation survival curve fitted to all the data from the three repeat experiments as a function of dose, between MCF-7-Bmi-1 cells (α=0.380±0.039 Gy^−1^) and MCF-7-pmscv cells (α=0.588±0.054 Gy^−1^) (P<0.05). Bmi-1 overexpression enhanced radiation resistance by SER=0.77 (SF_2_=0.257 for MCF-7-pMSCV cells; SF_2_=0.333 for MCF-7-Bmi-1 cells).

Next, we asked whether silencing of Bmi-1 expression by RNA interference (RNAi) was able to increase the sensitivity of MCF-7 cells to irradiation. As expected, Bmi-1 shRNA-expressing cells (MCF-7-KD cells) exhibited significantly enhanced radiosensitivity compared with control vector infected cells (MCF-7-Pre cells) (F=4.183, P<0.001; ANOVA test) (Fig. 1C and D). Statistical analysis of the survival parameters calculated using the linear-quadratic model also revealed a significant sensitization effect. The α value for MCF-7-KD and MCF-7-Pre cells was 0.814±0.056 and 0.580±0.041 Gy^−1^, respectively. Bmi-1 inhibition enhanced radiosensitivity by SER=1.40 (SF_2_=0.264 for MCF-7-Pre cells; SF_2_=0.189 for MCF-7-KD cells). Thus, these data suggested that Bmi-1 might be a critical regulator in radiation response in MCF-7 cells.

### Increased DNA double strand break (DSB) and decreased DSB repair caused by Bmi-1 silencing

**Figure 2:**
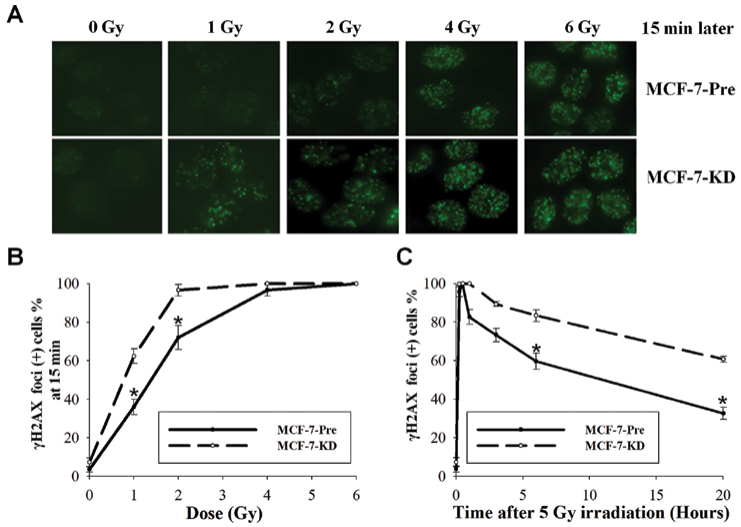
Bmi-1 knockout delayed DNA damage repair in MCF-7 cell line. (A) Graph showing γH2AX foci in Bmi-1-depleted MCF-7 cells vs. control 5 min later after irradiation. Original magnification, ×600. (B) γH2AX foci positive cells after different dose irradiation. Mean ± SD of three experiments are reported. (C) γH2AX foci positive cells at different time point after 5 Gy irradiation between MCF-7-control cells and MCF-7-knockout cells. ^*^P<0.001; t-test.

Phosphorylated histone H2AX (γH2AX) foci measurement, a more sensitive detecting method of DNA DSBs, was used to verify further this increase DNA DSBs after Bmi-1 silencing. As shown in Fig. 2A and B, by 15 min after 1 Gy, 36.0% of MCF-7-Pre cells demonstrated γH2AX foci compared with 62.3% of MCF-7-KD cells (P=0.001; t-test). At 2 Gy, 72.0% of MCF-7-Pre cells and 94.7% of MCF-7-KD cells displayed γH2AX foci (P=0.005; t-test). Foci were no difference in 4 Gy and observed in 100% of both cells at 6 Gy. These data further demonstrated that Bmi-1 knockdown enhanced DNA DSBs.

We next examined the kinetics of γH2AX in MCF-7-Pre and MCF-7-KD cells after irradiation. Fifteen minutes after 5 Gy, 95.7% of MCF-7-Pre cells and 100% of MCF-7-KD cells retained γH2AX foci (Fig. 2C). At 1 h, foci remained in 82% of MCF-7-Pre cells and 100% of MCF-7-KD cells. At 6 h, foci persisted in 59.7% of MCF-7-Pre cells and in 83.3% of MCF-7-KD cells (P=0.001; t-test). After 20 h, the number of foci for MCF-7-Pre and MCF-7-KD cells was 32.7 and 60.7%, respectively (P<0.001; t-test). The results showed that the residual number of γH2AX foci in MCF-7-KD cells was significantly higher than that of MCF-7-Pre cells after irradiation. Thus, DSB repair capacity in MCF-7-Pre and MCF-7-KD cells was significantly different, indicating that Bmi-1 silencing deceased DSB repair capacity.

### Bmi-1 knockdown promotes sub-G1 population and induces loss of mitochondrial membrane potential in irradiated MCF-7 cells

**Figure 3:**
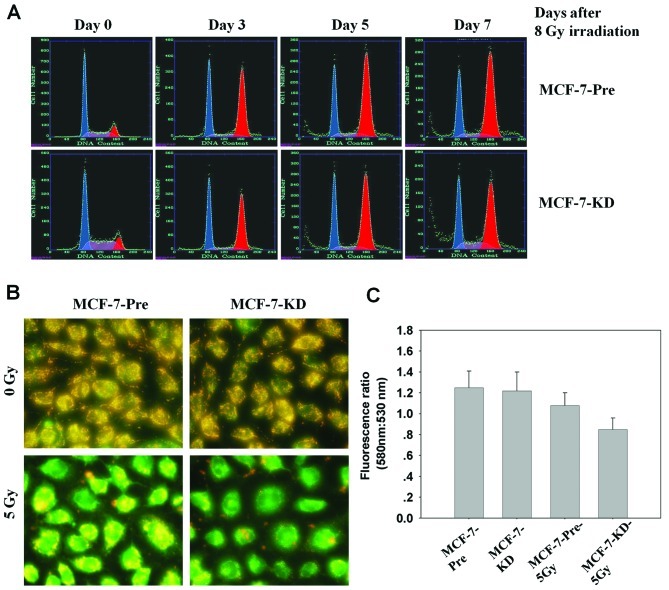
Bmi-1 depletion increased sub-G1 population. (A) Analysis of cell cycle distribution by flow cytometry. (B) Bmi-1 knockdown decreases mitochondrial membrane potential. Healthy mitochondria when it aggregates signal from JC-1 is depicted in red; when mitochondrial membrane potential collapses, signal corresponds to the green. Original magnification, ×400. (C) Quantitative results of fluorescence ratio (580 nm/530 nm).

It has reported that Bmi-1 enhanced cell survival by altering cell cycle regulatory protein expression and inhibiting apoptosis (34). Thus, we investigate cell cycle distribution in MCF-7-Pre and MCF-7-KD cells after 8 Gy irradiation in different time points by FACS. Radiation induced G1-phase and G2-phase cell cycle arrest in both MCF-7-Pre and MCF-7-KD cells, but there was no significant difference between them. However, the percentage of the sub-G1 population, which is indicative of apoptosis, significant increased in MCF-7-KD cells (11.3±1.1%) compared with MCF-7-Pre cells (6.6±0.8%, P<0.001; ANOVA) (Fig. 3A). The data indicated that Bmi-1 knockdown was able to induce apoptosis in MCF-7 cells.

Next, we examined whether Bmi-1 inhibtion induced apoptosis in MCF-7 cells could be coincident with changes in mitochondrial membrane potential (ΔΨm). We used the fluorescent dye, JC-1, as an indicator of the energy state of the mitochondria. A loss in mitochondria membrane potential is indicated by a decrease in red/green fluorescence intensity ratio. As shown in Fig. 3B and C, Bmi-1 inhibition led to a rapid drop in mitochondrial energy, as reflected by a fluorescence shift from green (emission 530 nm) to red (emission 580 nm) by 24 h after 5 Gy irradiation. These data demonstrated that apoptosis induced by Bmi-1 inhibition combined radiation may be closely related to mitochondrial function.

### Bmi-1 knockdown changes the signal pathway involved in apoptosis in MCF-7 cells

**Figure 4:**
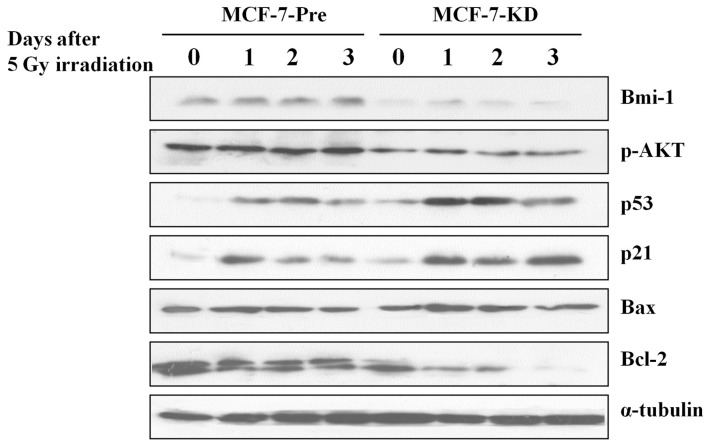
Western blot analysis of apoptosis related proteins. MCF-7-control cells and MCF-7-knockout cells were harvested on Days 0, 1, 2 and 3 after 5 Gy irradiation. Lysates were subjected to Western blot analysis with the labeled antibodies.

Then, we examined the levels of several apoptosis related proteins in MCF-7-Pre and MCF-7-KD cells. Western blot analysis showed that dramatically increased expression of p53, p21 and Bax protein, and decreased expression of p-AKT and Bcl-2 in irradiated MCF-7-KD cells compared with those in irradiated MCF-7-Pre cells (Fig. 4). This data indicated that Bmi-1 depletion sensitizes radiotherapy through activating apoptosis pathway.

## Discussion

In this report, we present the first evidence that Bmi-1 was correlated with radiation response in MCF-7 breast cancer cells. We showed that Bmi-1 overexpression developed radiation resistance, whereas Bmi-1 inhibition significantly increased radiation sensitivity with a DER of 1.40 by radiation clonogenic survival assay. It suggested that Bmi-1 might play an important role in the regulation of cellular response to radiation in MCF-7 cells. Thus, the results underscore the importance of Bmi-1 targeting in combination with irradiation in tumor therapy. In our previous study, we showed that Bmi-1 could enhance the invasion and metastasis of human breast cancer and predicts poor survival, Bmi-1 silencing reverses the expression of EMT markers and inhibits the Akt/GSK3β/Snail pathway (30). EMT promotes radioresistance in human tumor cells (28,29), down-regulation of Bmi-1 could be a novel strategy to sensitize radiotherapy by reversing EMT in human breast cancer.

It is well known that DSBs are suggestive of critical lesions in DNA caused by ionizing radiation. DSB is the main mechanism of tumor cell death after irradiation, and some protein involve in DSB could be used as a prognostic predictor for cancer (35). The major cause of radiotherapy failure is the success of DSB repair in tumor cells, leading to prolonged tumor cell survival. Molecules that are involved in DSB repair may be potential prognostic markers for the prediction of radiotherapy outcome, and hence, for optimization of treatment. We found that Bmi-1 inhibition dramatically increased DSB and significantly decreased the rate of DNA DSB recovery induced by irradiation, suggesting a participation of Bmi-1 in DNA strand damage and repair. Studies have showed that increased p-Akt had been linked to decreased radiation responsiveness and inhibition of p-Akt have radiosensitizing effects in various malignancies (36–39). Our study showed that p-Akt decreased after Bmi-1 inhibition, this might be one of reasons resulting in increased DSBs and decreased DSB repair in Bmi-1 knockdown cells.

If complete DNA damage repair fails, apoptosis is triggered for the elimination of damaged cells. We noticed that the number of apoptotic cells in Bmi-1 knockdown cells was much higher compared with that in the controls using FACS analysis. The mechanism of Bmi-1 inhibition combined radiation induced apoptosis is complex. A possible reason is that mitochondria-dependent pathway for apoptosis was activated in response to combined Bmi-1 inhibition with ionizing radiation. In many tumor models of apoptosis, oxidative stress induced by ROS is a frequent mediator of apoptosis and produced massive cellular damage associated with lipid peroxidation, loss of mitochondrial membrane potential (ΔΨm), deletion of cellular antioxidants and Bax translocation to mitochondrial (40–43). In this study, we found MCF-7 cell silencing of Bmi-1 seems to be more susceptible to cell death with loss of ΔΨm induced by irradiation (Fig. 3B and C). The underlying mechanism by which Bmi-1 inhibition induces loss of ΔΨm in MCF-7 cells remains undefined. Several mitochondrial proteins, including Bcl-2 and Bax, possible play important roles in the process. During apoptosis, the imbalance between the expression of anti- and pro-apoptotic proteins is important. Our data showed that Bmi-1 inhibition enhanced proapototic p53, p21 and Bax expression but decreased levels of the antiapoptotic proteins p-AKT and Bcl-2 expression compared with control cells following irradiation.

In conclusion, we report the enhanced radiosensitivity of a breast cancer cell line after Bmi-1 inhibition using shRNA. The increased sensitivity was associated with increased DSBs and decreased DSB repair. We also showed that combination Bmi-1 inhibition with irradiation could induce apoptosis, collapse of mitochondrial membrane potential, elevated p53, p21, Bax expresseion, and deceased Bcl-2 expression. Our results suggest that Bmi-1 inhibition play an additive effect to radiation therapy in MCF-7 mammary carcinoma cells providing a novel target for radio-sensitizing breast cancer.

## Abbreviations

Bmi-1: B-cell-specific moloney murine leukemia virus integration site 1
EMT: epithelial-mesenchymal transition
DSB: DNA double strand break
KD: knock down
GSK3β: glycogen synthase kinase 3β
Akt: protein kinase B
